# Changes in endotracheal tube cuff pressure in mechanically ventilated adult patients

**DOI:** 10.1186/2052-0492-2-7

**Published:** 2014-01-31

**Authors:** Asuka Motoyama, Shota Asai, Hiroyuki Konami, Yuri Matsumoto, Takuyo Misumi, Hideaki Imanaka, Masaji Nishimura

**Affiliations:** The University of Tokushima Graduate School, 2-50-1 Kuramotocho, 770-8503 Tokushima, Tokushima Prefecture, Japan; Critical Care Medicine, Kobe University Hospital, 1-1 Rokkodaicho, Nada Ward, Kobe, Hyogo Prefecture, 657-0013 Japan; Emergency and Disaster Medicine, Tokushima University Hospital, 2-50-1 Kuramotocho, Tokushima, Tokushima Prefecture, 770-8503 Japan; Emergency and Critical Care Medicine, Tokushima University Hospital, 2-50-1 Kuramotocho, Tokushima, Tokushima Prefecture, 770-8503 Japan

**Keywords:** Cuff pressure, Endotracheal tube, Critically ill patients, Mechanical ventilation

## Abstract

During mechanical ventilation, endotracheal tube cuff pressure should be maintained within proper range. We investigated the effect of frequent adjustment on cuff pressure in 27 mechanically ventilated patients. Cuff pressure was recorded every 2 h and was adjusted to 24 cmH_2_O each time. We found that cuff pressure was decreased by 4.9 ± 2.9 cmH_2_O from the target value. Cuff pressure decreased to less than 20 cmH_2_O in 45% of measurement occasions 2 h after adjusting it to 24 cmH_2_O.

## Correspondence

To prevent gas leakage and aspiration, an endotracheal tube (ETT) with a cuff is generally used for mechanically ventilated patients. Because excessive cuff pressure decreases tracheal capillary perfusion, and insufficient cuff pressure leads to aspiration of oropharyngeal contents, [1–3] cuff pressure should be maintained within the proper range. Cuff pressure measurements are routinely taken every 8 to 24 h, and during the interval, air inside the cuff may escape from the ETT cuff surface or through the pilot balloon valve. It remains unknown whether, through frequent adjustment, cuff pressure can be maintained within the target range. We prospectively collected 1,846 data points of cuff pressure from 27 adult patients receiving mechanical ventilation for longer than 48 h with cuffed ETTs, standard high-volume low-pressure cuff (Hi-Lo Mallinckrodt Medical, Dublin, Ireland). Nurses measured the cuff pressure every 2 h using a cuff inflator (Cuff Pressure Gauge, VBM Medizintechnik GmbH, Baden-Württemberg, Germany) each time readjusting the pressure to 24 cmH_2_O. Ventilatory settings and body position remained unchanged during the 2-h interval. The study was approved by the hospital research board, which waived the requirement for informed consent for this observational study that was part of routine care.

**Figure 1:**
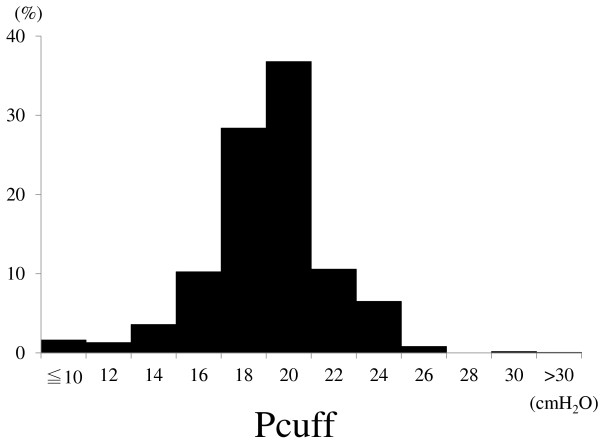
**Distribution of cuff pressure.**

Cuff pressure deviation from the target value (24 cmH_2_O) was −4.9 ± 2.9 cmH_2_O. Cuff pressure was below 20 cmH_2_O in 45% of the measurements, below 24 cmH_2_O in 93%, and over 30 cmH_2_O in 0.05% (Figure 1). Loss of cuff pressure is known to increase the risk of complications cuff pressure below 20 cmH_2_O is associated with the development of ventilator-associated pneumonia [1, 2, 4, 5]. Nseir et al. [3], measuring cuff pressure every 8 h, found that cuff pressure was maintained within recommended range (20 to 30 cmH_2_O) in only 18% of patients, that it was lower than 20 cmH_2_O at least once for 54% of patients, and that it was over 30 cmH_2_O at least once for 73% of patients. In our study, the cuff tended to deflate, probably owing to different patient characteristics than in Nseir’s study.

Because frequent readjustment did not prevent cuff pressure loss, we suspected that the measurement procedure itself might contribute to changes in cuff pressure. The air compressed in the cuff might escape to the measurement system during the connection procedure. Further study is needed to clarify the effect of measurement procedures in a broader variety of situations. Our study has several limitations: small population, varied observation time, and lack of evaluation of clinically significant outcomes. In conclusion, cuff pressure decreased to less than 20 cmH_2_O in 45% of measurement occasions taken from critically ill patients 2 h after adjusting it to 24 cmH_2_O.

## Abbreviations

ETT: endotracheal tube.
